# The bright and the dark sides of L-carnitine supplementation: a systematic review

**DOI:** 10.1186/s12970-020-00377-2

**Published:** 2020-09-21

**Authors:** Angelika K. Sawicka, Gianluca Renzi, Robert A. Olek

**Affiliations:** 1 Department of Human Physiology, Faculty of Health Sciences, Medical University of Gdansk, 80-210 Gdansk, Poland; 2 University of Camerino, 62032 Camerino, Italy; 3 Department of Athletics, Strength and Conditioning, Poznan University of Physical Education, Krolowej Jadwigi 27/39, 61-871 Poznan, Poland

**Keywords:** Insulin-like growth factor-1, Protein kinase B, Mammalian target of rapamycin, Forkhead box O, MuRF-1, Atrogin-1, Trimethylamine-N-oxide

## Abstract

**Background:**

L-carnitine (LC) is used as a supplement by recreationally-active, competitive and highly trained athletes. This systematic review aims to evaluate the effect of prolonged LC supplementation on metabolism and metabolic modifications.

**Methods:**

A literature search was conducted in the MEDLINE (via PubMed) and Web of Science databases from the inception up February 2020. Eligibility criteria included studies on healthy human subjects, treated for at least 12 weeks with LC administered orally, with no drugs or any other multi-ingredient supplements co-ingestion.

**Results:**

The initial search retrieved 1024 articles, and a total of 11 studies were finally included after applying inclusion and exclusion criteria. All the selected studies were conducted with healthy human subjects, with supplemented dose ranging from 1 g to 4 g per day for either 12 or 24 weeks. LC supplementation, in combination with carbohydrates (CHO) effectively elevated total carnitine content in skeletal muscle. Twenty-four-weeks of LC supplementation did not affect muscle strength in healthy aged women, but significantly increased muscle mass, improved physical effort tolerance and cognitive function in centenarians. LC supplementation was also noted to induce an increase of fasting plasma trimethylamine-N-oxide (TMAO) levels, which was not associated with modification of determined inflammatory nor oxidative stress markers.

**Conclusion:**

Prolonged LC supplementation in specific conditions may affect physical performance. On the other hand, LC supplementation elevates fasting plasma TMAO, compound supposed to be pro-atherogenic. Therefore, additional studies focusing on long-term supplementation and its longitudinal effect on the cardiovascular system are needed.

## Background

The main function of L-carnitine (LC) is the transport of long-chain fatty acids into the mitochondrial matrix for their conversion in energy, via β-oxidation process [1]. Moreover, LC by the reaction with acetyl-CoA and maintaining the acetyl-CoA/CoA ratio in the cell regulates pyruvate dehydrogenase activity [2]. LC also plays an important role in the regulation of metabolic pathways involved in skeletal muscle protein balance: proteolysis and protein synthesis [3]. Furthermore, LC acts as anti-oxidant and anti-inflammatory compound [3]; thus, it may attenuate the exercise-induced muscle damage.

The opinion that LC supplementation does not change metabolism is based mostly on short-term supplementation protocols [4]. Recent studies demonstrate that prolonged supplementation, especially in combination with carbohydrates (CHO), may increase muscle total carnitine (TC) content in skeletal muscle [5–7]. Therefore, LC supplementation in specific conditions may affect physical performance. On the other hand, LC has been proposed as the red meat nutrient responsible for atherosclerosis promotion [8]. As a potential link between red meat consumption and the increasing risk of cardiovascular disease, trimethylamine-N-oxide (TMAO) has been indicated [8]. Since LC is still used by the athletes [9, 10], the aim of this systematic review is to evaluate the effect of prolonged LC supplementation on metabolism/metabolic changes in healthy human subjects.

## Methods

### Eligibility criteria

The PICOS strategy was defined as follows: “P” (participants) human subjects, “I” (interventions) oral LC treatment, “C” (comparisons) between supplementation and placebo, supplementation and control, or pre- and post- supplementation, “O” (outcomes) muscle variables, and “S” (study design) randomized controlled trials, non-randomized controlled trials, non-randomized non-controlled trials.

Studies with the following criteria were excluded: described in languages other than English, articles without full-text availability, reviews and case reports. Subsequently, the following eligibility criteria were applied: a) healthy human subjects; b) supplementation at least for 12 weeks; c) oral LC administration; d) no drugs co-ingestion; e) no multi-ingredients supplementation.

### Information sources and search

The literature was explored using the MEDLINE (via PubMed) and Web of Science databases, including all articles published from the inception up February 2020. The search was conducted using the terms: “carnitine supplementation” or “carnitine treatment” in combination with “exercise”, “training”, “athletic performance”, “muscle strength”, “muscle fatigue”, “muscle damage”, “muscle recovery”, “muscle synthesis” or “proteolysis”.

### Study selection

Firstly, studies were assessed by title verification between databases (duplicates were removed). The second assessment performed by abstracts analysis, excluded studies in a language other than English, studies with lack of full text, reviews, case reports, animal studies and in-vitro studies. The last step was performed by analysis of full manuscripts based on the described above eligibility criteria.

### Data collection process

The following information was compiled for each study: authors, year of publication, type of study, length of supplementation, a dose of supplementation and main effect. Lastly, the thematic analysis was carried out, to synthesize and interpret all the data that appeared from the included publications. The process of selecting papers, data collection as well as the quality assessment was performed independently by two authors (A.S., G.R.), and all disagreements were resolved by the discussion with the third author (R.O).

## Results

### Study selection

**Fig. 1:**
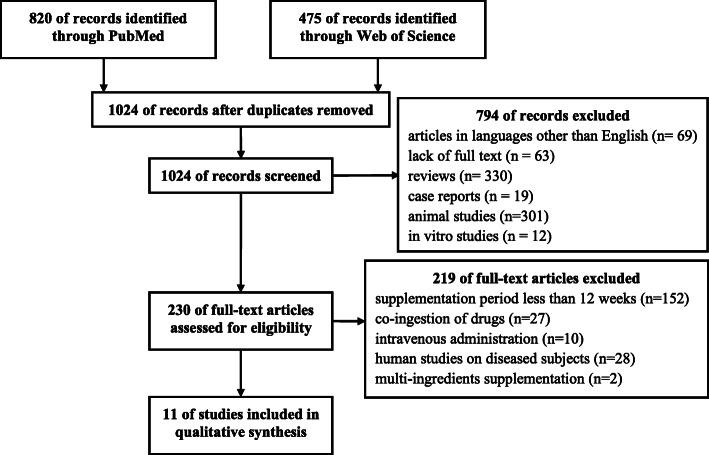
Flowchart on the search and selection of articles included in the review

By the above-described search strategy, 1295 publications were identified. After the first selection, adjusted by duplicates, persisted 1024 articles. Of these, 794 were excluded after abstracts screening and identified articles in languages other than English, lack of full text or being review articles, case reports, animal or in-vitro studies. The full texts of 230 articles were screened by eligibility criteria. Finally, to the qualitative analysis were accepted 11 studies performed on healthy human subjects, treated for at least 12 weeks with LC administered orally, with no drugs or any other multi-ingredient supplements co-ingestion (Fig. 1).

### Description of the included studies

Table 1 provides details and results of the 11 studies reviewed. Selected studies were published between 2002 and 2020. In the selected studies, participants were supplemented in a dose ranging from 1 g to 4,5 g per day for either 12 or 24 weeks, mostly by L-carnitine-L-tartrate (LCLT). In three studies, supplementations were combined with carbohydrates (CHO) [5–7], and in one with L-leucine [18].

**Table 1:** Summary and results of the studies reviewed examining the LC supplementation

Studies	Participants characteristics	Study design	Supplementation dose and period	Main effect
[11]	Moderately trained male subjects (*n* = 7) age 23–25	NRNC	4 g LC/day for 3 months	Increase of TC plasma concentration after the supplementation; No change in muscle TC concentration, mitochondrial enzymes activity, physical performance and muscle fiber composition
[12]	Male vegetarians (*n* = 16) and omnivores (C) (*n* = 8) age 18–40	NRC	2 g LCLT /day for 12 weeks	Increase of TC plasma concentration after the supplementation and muscle TC concentration only in vegetarians; No change in physical performance and muscle metabolism either in omnivores or vegetarians.
[13]	Middle aged untrained male subjects (S *n* = 12; P *n* = 12) age not reported (both groups involved in endurance training; 3x/week)	RC	2 g LCLT /day for 12 weeks	Increase of TC plasma concentration after the supplementation; Plasma triacylglycerols and free fatty acids not affected by training or supplementation; Training resulted in an increase in the mRNA expression of genes coding proteins involved in long chain fatty acid transport in white blood cells, LC supplementation enhanced the effect on gene expression
[6]	Non-vegetarian, male recreational athletes (S n = 6; P *n* = 6) age 28 ± 2 (S); 25 ± 2 (P)	RC	2 g LCLT + 80 g CHO /day for 12 weeks	Increase in muscle TC concentration after LC supplementation; Upregulation of seventy-three genes relating to fuel metabolism in LC vs. control; Higher exercise energy expenditure after LC supplementation; No change in carnitine palmitolytransferase 1 activity; Body mass and whole-body fat mass increased in control, but did not change in LC supplemented
[5]	Non-smoking, non-vegetarian recreational athletes (S *n* = 7; P *n* = 7) age 26 ± 2	RC	2 g LCLT + 80 g CHO /day for 24 weeks	Increase in muscle TC concentration after LC supplementation; Lower muscle glycogen utilization during low intensity exercise, lower lactate production during high intensity exercise, higher work output during a 30 min ‘all-out’ exercise performance test in LC supplemented group;
[7]	Healthy, non-vegetarian, untrained males (S *n* = 7; P *n* = 7) age 23 ± 2 (both groups involved in HIIT; 3x/week)	RC	2.25 g LCLT + 80 g CHO /day for 24 weeks	Muscle TC concentration tend to increase after LC supplementation (*p* = 0.06 vs. pre-supplementation); Skeletal muscle adaptations to training not augmented by elevated muscle carnitine availability;
[14]	Centenarians (S *n* = 27; P *n* = 27) age 100–106	RC	2 g LC/day for 24 weeks	Increase of TC plasma concentration after the supplementation; Fat mass reduction, muscle mass elevation, physical effort tolerance and cognitive function improvement in LC supplemented group
[15]	Healthy women (S *n* = 11; P *n* = 9) age 65–70	RC	1.5 g LCLT /day for 24 weeks	Increase of free carnitine plasma concentration after the supplementation; No changes in body composition, skeletal muscle strength and IGF-1 after LC supplementation
[16]	Healthy women (S *n* = 11; P *n* = 9) age 65–70	RC	1.5 g LCLT /day for 24 weeks	Increase of plasma TMAO concentration after the supplementation; No changes in serum C-reactive protein, interleukin-6, tumor necrosis factor-α, L-selectin, P-selectin, vascular cell adhesion molecule-1, intercellular adhesion molecule-1 and lipid profile after LC supplementation
[17]	Healthy women (S *n* = 11; P *n* = 9) age 65–70	RC	1.5 g LCLT /day for 24 weeks	No changes in plasma GBB or serum ox-LDL, myeloperoxidase, protein carbonyls, homocysteine, and uric acid concentrations
[18]	Healthy aged women (S *n* = 12; P *n* = 13; C *n* = 12) age 67 ± 3 (all groups involved in resistance training 3x/week)	RC	1 g LCLT + 3 g L-leucine/day for 24 weeks	Increase of plasma TMAO concentration after the supplementation; Increase of D-loop methylation in platelets of LC supplemented

Groups: *C* control; *S* supplemented; *P* placebo; Study design: *RC* randomized controlled; *NRC* non-randomized controlled; *NRNC* non-randomized non-controlled; *LCLT* L-carnitine-L-tartrate; *HIIT* high-intensity interval training

Muscle carnitine content was not affected following 12 weeks of LC supplementation alone [11, 12]. On the other hand, LC supplementation in combination with CHO effectively elevated muscle TC after 12 [6] and 24 weeks [5]. Moreover, 12 weeks of supplementation alone [13], or in combination with CHO [6] promote the expression of the genes related to fatty acids and carnitine metabolism.

Twenty-four-weeks of LC supplementation alone did not affect muscle strength in healthy aged women [15], but significantly increased muscle mass, improved physical effort tolerance and cognitive function in centenarians [14].

In two studied groups of healthy aged woman, LC supplementation alone [16, 17], or in combination with L-leucine [18], induced an increase of fasting plasma TMAO levels. However, higher TMAO was not associated with determined inflammatory [16] nor oxidative stress [17] markers. Moreover, despite elevated TMAO, LC supplementation together with resistance training induced positive changes in mitochondrial DNA methylation of platelets [18].

## Discussion

The present findings have been debated in the six separate paragraphs, and for a better picture of LC supplementation, other studies were also disputed.

### “Fat burner”

It has been assumed that LC supplementation, by increasing muscle carnitine content, optimizes fat oxidation and consequently reduces its availability for storage [19]. Nevertheless, the belief that carnitine is a slimming agent has been negated in the middle of 90s [20]. Direct measurements of carnitine in skeletal muscles failed to show any elevation in the muscle carnitine concentration following 14 days of 4 g/day [21], or 6 g/day [22] LC ingestion. These findings implied that LC supplementation was not able to increase fat oxidation and improve exercise performance by the proposed mechanism. Indeed, many original investigations, summarized in later review [4], indicated that LC supplementation lasting up to 4 weeks, neither increase fat oxidation nor improve performance during prolonged exercises.

Since LC concentration in skeletal muscles is higher than that of blood plasma, active uptake of carnitine must take place [23]. Stephens et al. [24] noted that 5 h steady-state hypercarnitinemia (~ 10-fold elevation of plasma carnitine) induced by the intravenous LC infusion does not affect skeletal muscle TC content. On the other hand, similar intervention in combination with controlled hyperinsulinemia (~ 150mIU/L) elevates TC in skeletal muscle by ~ 15% [24, 25]. Moreover, higher serum insulin maintained by the consumption of simple sugars resulted in augmented LC retention in healthy human subjects supplemented by LC for 2 weeks [26]. Based on these results, Authors suggested that oral ingestion of LC, combined with CHO for activation carnitine transport into the muscles, should take ~ 100 days to increase muscle carnitine content by ~ 10% [26]. This assumption has been confirmed in later studies [5–7]. These carefully conducted studies clearly showed that prolonged procedure (for ≥12 weeks) of a daily LC and CHO ingestion induced a raise of skeletal muscle TC levels [5–7], affecting exercise metabolism [5], improving performance [5] and energy expenditure [6], without altering body composition [6]. The lack of body fat stores loss may be explained by the 18% increase in body fat mass associated with CHO supplementation alone, noted in the control group [6].

Nevertheless, 12 weeks of LC supplementation 2 g/day applied without CHO, elevated muscle TC only in vegetarian but not in omnivores [12]. Neither exercise metabolism nor muscle metabolites were modified by augmented TC in vegetarian [12].

### Skeletal muscle protein balance regulation

Skeletal muscle mass depends on the rates of protein synthesis and degradation. Elevated protein synthesis and attenuated proteolysis are observed during muscle hypertrophy. Both of these processes are mainly regulated by the signaling pathway: insulin-like growth factor-1 (IGF-1) – phosphoinositide-3-kinase (PI3K) – protein kinase B (Akt) – mammalian target of rapamycin (mTOR). The activation of mTOR leads to phosphorylation and activation of S6 kinases (S6Ks) and hyperphosphorylation of 4E-binding proteins (4E-BPs), resulting in the acceleration of protein synthesis. At the same time, Akt phosphorylates and inactivates forkhead box O (FoxO), thereby inhibit the responsible for proteolysis ubiquitin ligases: muscle-specific RING finger-1 (MuRF-1) and muscle atrophy F-box protein (atrogin-1), (for review see [27–29]).

**Fig. 2:**
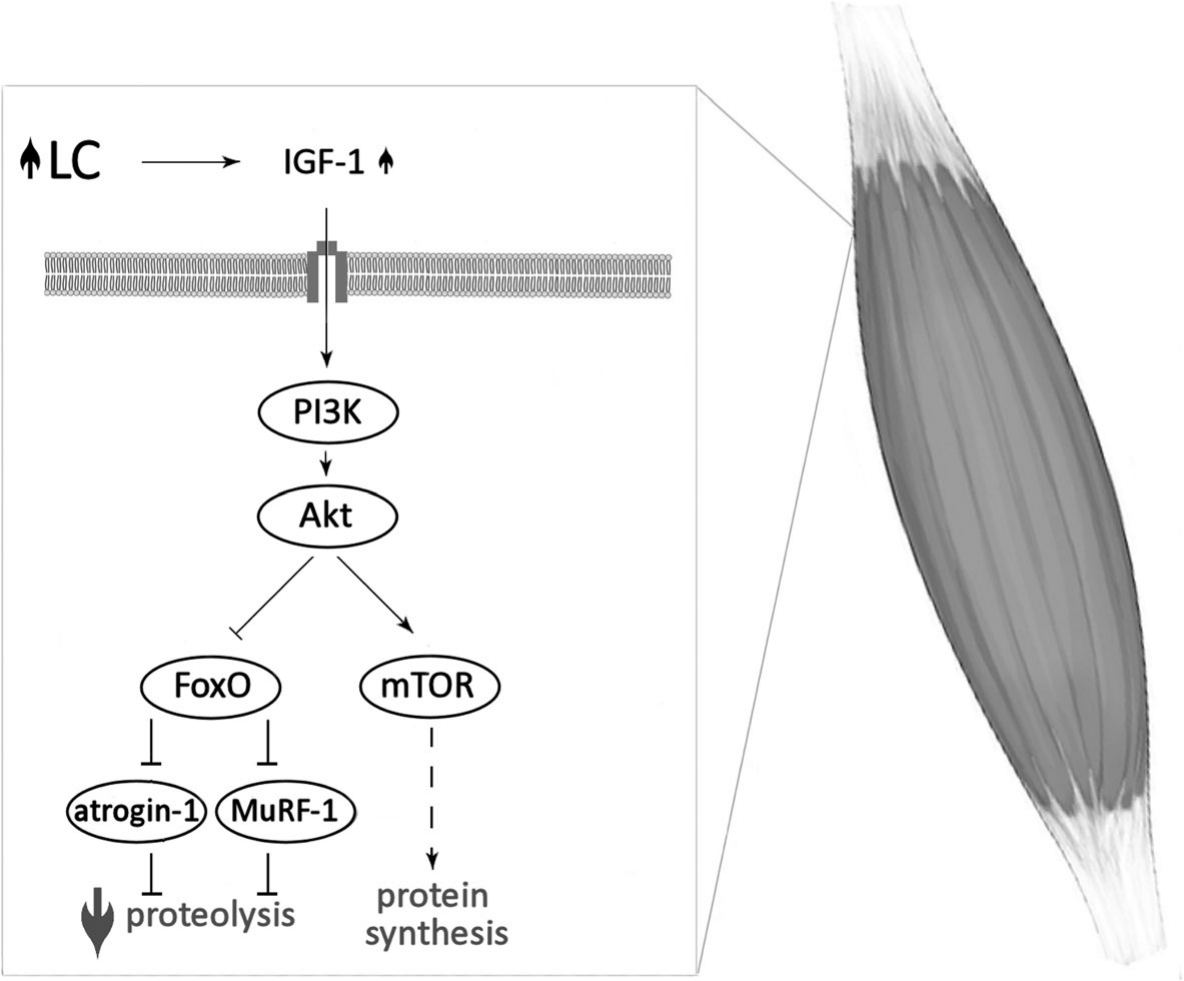
The association between LC supplementation and the regulation of metabolic pathways involved in muscle protein balance. L-carnitine (LC); insulin-like growth factor-1 (IGF-1); phosphoinositide-3-kinase (PI3K); protein kinase B (Akt); mammalian target of rapamycin (mTOR); forkhead box O (FoxO); muscle-specific RING finger-1 (MuRF-1); muscle atrophy F-box (atrogin-1); increase (); decrease (); activation (); inactivation ()

The association between LC supplementation and the regulation of metabolic pathways involved in muscle protein balance have been shown in several animal studies (Fig. 2) [30–35]. Four weeks of LC supplementation in rats increased plasma IGF-1 concentration [33]. Elevated circulating IGF-1 led to an activation of the IGF-1–PI3K–Akt signalling pathway, causing augmented mTOR phosphorylation and higher phospho-FoxO/total FoxO ratio in skeletal muscle of LC supplemented rats [33]. FoxO inactivation attenuated MURF-1 expression in *quadriceps fem*oris muscle of supplemented rats (compared to control) [33]. Moreover, LC administrated for 2 weeks suppresses atrogin-1 messenger RNA (mRNA) level in suspended rats’ hindlimb [35], and only 7 days of LC administration downregulates MuRF-1 and atrogin-1 mRNAs reducing muscle wasting in a rat model of cancer cachexia [32]. All these findings together might suggest that LC supplementation protect muscle from atrophy, especially in pathophysiological conditions.

In fact, administration of acetyl-L-carnitine 3 g/day for 5 months in HIV-seropositive patients induced ten-fold increase in serum IGF-1 concentration [36]. Conversely, neither 3 weeks LC supplementation in healthy, recreationally weight-trained men [37], nor 24 weeks LC supplementation in aged women [15] did not affect circulating IGF-1 level concentration. Various effects might be due to different IGF-1 levels; significantly lower in the HIV-seropositive patients than in healthy subjects [38]. Additionally, 8 weeks of LC supplementation in healthy older subjects, did not change total and phosphorylated mTOR, S6K and 4E-BP proteins level of *vastus lateralis* muscle [39]. It must be highlighted that rat skeletal muscle TC increases ~ 50–70% following 4 weeks of LC supplementation [33, 34], whereas comparable elevation has never been observed in human studies, even after 24 weeks of supplementation [5, 7].

### Body composition

These findings altogether suggest that prolonged LC supplementation might affect body composition in specific conditions.

### Obesity

A recent meta-analysis, summarized studies focused on LC supplementation for a prolonged time (median 3 months) [40]. Pooled results demonstrated a significant reduction in weight following LC supplementation, but the subgroups analysis revealed no significant effect of LC on body weight in subjects with body mass index (BMI) below 25 kg/m^2^. Therefore, authors suggested that LC supplementation may be effective in obese and overweight subjects. Surprisingly, intervention longer than 24 weeks showed no significant effect on BMI [40].

### Training

It has been assumed that a combination of LC supplementation with increased energy expenditure may positively affect body composition. However, either with aerobic [41, 42] or resistance [43] training, LC supplementation has not achieved successful endpoint. Six weeks of endurance training (five times per week, 40 min on a bicycle ergometer at 60% maximal oxygen uptake) together with LC supplementation (4 g/day) does not induce a positive effect on fat metabolism in healthy male subjects (% body fat 17.9 ± 2.3 at the beginning of the study) [41]. Similarly, lack of LC effect has been reported in obese women [42]. Eight weeks of supplementation (2 g/day) combined with aerobic training (3 sessions a week) had no significant effects on body weight, BMI and daily dietary intake in obese women [42].

In the recent study, LC supplementation 2 g/day has been applied in combination with a resistance training program (4 days/week) to healthy men (age range 18–40 y.o.), for 9 weeks [43]. Body composition, determined by dual energy X-ray absorptiometry, indicated no significant effect in fat mass and fat-free mass due to supplementation. Moreover, LC administration did not influence bench press results. The number of leg press repetitions and the leg press third set lifting volume increased in the LC group compared to the placebo group [43]. Different LC effect in the limbs may be associated with the higher rates of glycogenolysis during arm exercise at the same relative intensity as leg exercise [44].

### Sarcopenia

Aged people have accelerated protein catabolism, which is associated with muscle wasting [45]. LC could increase the amount of protein retention by inhibition of the proteolytic pathway. Six months of LC supplementation augmented fat free mass and reduced total body fat mass in centenarians [14]. Such effect was not observed in elder women (age range 65–70 y.o.) after a similar period of supplementation [15]. The effectiveness of LC supplementation may result from the age-wise distribution of sarcopenia. The prevalence of sarcopenia increased steeply with age, reaching 31.6% in women and 17.4% in men older than 80 years [46]. In subjects below 70 years presarcopenia, but not sarcopenia symptoms were noted [46].

### Oxidative imbalance and muscle soreness

Muscle damage may occur during exercise, especially eccentric exercise. In the clearance of damaged tissues assist free radicals produced by neutrophils. Therefore, among other responses to exercise, neutrophils are released into the circulation. While neutrophil-derived reactive oxygen species (ROS) play an important role in breaking down damaged fragments of the muscle tissue, ROS produced in excess may also contribute to oxidative stress (for review see [47, 48].

Based on the assumption that LC may provide cell membranes protection against oxidative stress [49], it has been hypothesized that LC supplementation would mitigate exercise-induced muscle damage and improve post-exercise recovery. Since plasma LC elevates following 2 weeks of supplementation [21, 22], short protocols of supplementation may be considered as effective in attenuating post-exercise muscle soreness. The findings indicated that 3 weeks of LC supplementation, in the amount 2-3 g/day, effectively alleviated pain [50–53]. It has been shown, through magnetic resonance imaging technique that muscle disruption after strenuous exercise was reduced by LC supplementation [37, 51]. This effect was accompanied by a significant reduction in released cytosolic proteins such as myoglobin and creatine kinase [50, 52, 53] as well as attenuation in plasma marker of oxidative stress - malondialdehyde [51, 53, 54]. Furthermore, 9 weeks of LC supplementation in conjunction with resistance training revealed a significant increase of circulating total antioxidant capacity and glutathione peroxidase activity and decrease in malondialdehyde concentration [43].

### Risks of TMAO

In 1984 Rebouche et al. [55], showed that rats, orally receiving radiolabeled LC, metabolized it to γ-butyrobetaine (up to 31% of the administered dose, present primary in feces) and TMAO (up to 23% of the administered dose, present primary in urine). On the contrary, these metabolites were not produced by the rats receiving the isotope intravenously and germ-free rats receiving the tracer orally, suggesting that orally ingested LC is in part degraded by the gut’s microorganisms [55]. Similar observations were noted in later human studies [56, 57], with the peak serum TMAO observed within hours following oral administration of the tracer [56]. Prolonged LC treatment elevates fasting plasma TMAO [16–18, 58, 59]. Three months of oral LC supplementation in healthy aged women induced ten-fold increase of fasting plasma TMAO, and this level remained elevated for the further 3 months of supplementation [16]. Four months after cessation of LC supplementation, plasma TMAO reached a pre-supplementation concentration, which was stable for the following 8 months [60].

In 2011 Wang et al. [61] suggested TMAO as a pro-atherogenic factor. Since diets high in red meat have been strongly related to heart disease and mortality [62], LC has been proposed as the red meat nutrient responsible for atherosclerosis promotion [8]. As a potential link between red meat consumption and the increasing risk of cardiovascular disease, TMAO has been indicated [8]. Numerous later studies have shown the association between increased plasma TMAO levels with a higher risk of cardiovascular events [63–66]. The recent meta-analyses indicated that in patients with high TMAO plasma level, the incidence of major adverse cardiovascular events was significantly higher compared with patients with low TMAO levels [67], and that all-cause mortality increased by 7.6% per each 10 μmol/L increment of TMAO [68].

Since red meat is particularly rich in LC [69], dietary intervention in healthy adults, indicated a significant increase in plasma and urine TMAO levels following 4 weeks of the red meat-enriched diet [70]. The rise of plasma TMAO was on average three-fold compared with white meat and non-meat diets [70]. Conversely, habitual consumption of red, processed or white meat did not affect plasma TMAO in German adult population [71]. Similarly, a minor increase in plasma TMAO was observed following red meat and processed meat consumption in European multi-center study [72].

In the previous century, the underlined function of TMAO was the stabilization of proteins against various environmental stress factors, including high hydrostatic pressure [73]. TMAO was shown as widely distributed in sea animals [74], with concentration in the tissue increasing proportionally to the depth of the fishes natural environment [75]. Consequently, fish and seafood nutritional intake has a great impact on TMAO level in the human body [76], significantly elevating also plasma TMAO concentration [72]. Therefore, link between plasma TMAO and the risk of cardiovascular disease [8] seems like a paradox, since more fish in the diet reduces this risk [77].

Not only dietary modification may affect TMAO plasma levels. Due to TMAO excretion in urine [56, 57], in chronic renal disease patients, TMAO elimination from the body fails, causing elevation of its plasma concentration [78]. Therefore, higher plasma TMAO in humans was suggested as a marker of kidney damage [79]. It is worthy to note that cardiovascular disease and kidney disease are closely interrelated [80] and diminished renal function is strongly associated with morbidity and mortality in heart failure patients [81]. Moreover, decreased TMAO urine excretion is associated with high salt dietary intake, increasing plasma TMAO concentration [82].

The relation between TMAO and chronic disease can be ambiguous, involving kidney function [79], disturbed gut-blood barrier [83], or flavin-containing monooxygenase 3 genotype [84]. Thus, whether TMAO is an atherogenic factor responsible for the development and progression of cardiovascular disease, or simply a marker of an underlined pathology, remains unclear [85].

### Adverse effects

Carnitine preparations administered orally can occasionally cause heart-burn or dyspepsia [86]. No adverse events associated with LC administration were recorded at a dose 6 g/day for 12 months of supplementation in the patients with acute anterior myocardial infarction [87], or at a dose 1.274 g/day (range 0.3–3 g/day) and duration 348 days (range 93–744 days) in patients with liver cirrhosis [88]. Summarizing the risk associated with LC supplementation Hathcock and Shao [89] indicated that intakes up to 2 g/day are safe for chronic supplementation.

Although the optimal dose of LC supplementation for myocardial infarction is 3 g/day in terms of all-cause mortality [90], even lower LC intake elevates fasting plasma TMAO [16–18, 58, 59], which is ten-fold higher than control after 3 months of supplementation [16, 17]. It is worthy to mention that Bakalov et al. [91] analyzing European Medicine Agency database of suspected adverse drug reaction, noticed 143 cases regarding LC.

## Strengths and limitations

The strength of this review is a focus on the period of LC treatment, very important aspect often missed in many articles dealing with this supplement. To date, only few studies have examined the effects of LC supplementation for at least 12 weeks, which is, on the other hand, the main limitation of the current review. This limitation is also magnified by the varied design of the studies available including different supplementation protocols and outcome measures. There is also a high degree of heterogeneity among participants of the analyzed studies. Therefore, the results should be taken with caution, and more research is required before definitive recommendations.

## Conclusions

Lasting for several years opinion that LC supplementation does not change metabolism, especially exercise metabolism, is based mostly on short-term supplementation protocols. Nevertheless, LC is still used by elite [9] and sub-elite [10] athletes. Recent studies suggest that LC supplementation may elevate muscle TC content; therefore, modify muscle fuel metabolism and performance during the exercise. Due to insulin-mediated LC transport to the muscle, oral administration regimen should be combined with CHO. Because of LC poor bioavailability, it is likely that the supplementation protocol would take at least 3 months. Shorter period of supplementation may be effective in prevention of exercise-induced muscle damage, but not metabolic changes.

On the other hand, it is also clear that prolonged LC supplementation elevates fasting plasma TMAO [16–18, 58, 59], compound supposed to be pro-atherogenic [61]. Therefore, additional studies focusing on long-term supplementation and its longitudinal effect on the TMAO metabolism and cardiovascular system are needed.

## Data Availability

Not applicable.

## Abbreviations

LC: L-carnitine
TC: Total carnitine
TMAO: Trimethylamine-N-oxide
CHO: Carbohydrates
IGF-1: Insulin-like growth factor-1
PI3K: Phosphoinositide-3-kinase
Akt: Protein kinase B
mTOR: Mammalian target of rapamycin
S6K: S6 kinase
4E-BP: 4E-binding protein
FoxO: Forkhead box O
MuRF-1: Muscle-specific RING finger-1
atrogin-1: Muscle atrophy F-box
mRNA: Messenger RNA
BMI: Body mass index
ROS: Reactive oxygen species

## References

[1] Bremer J (1983). Carnitine--metabolism and functions. Physiol Rev.

[2] Arenas J, Huertas R, Campos Y, Diaz AE, Villalon JM, Vilas E (1994). Effects of L-carnitine on the pyruvate dehydrogenase complex and carnitine palmitoyl transferase activities in muscle of endurance athletes. FEBS Lett.

[3] Ringseis R, Keller J, Eder K (2013). Mechanisms underlying the anti-wasting effect of L-carnitine supplementation under pathologic conditions: evidence from experimental and clinical studies. Eur J Nutr.

[4] Brass EP (2000). Supplemental carnitine and exercise. Am J Clin Nutr.

[5] Wall BT, Stephens FB, Constantin-Teodosiu D, Marimuthu K, Macdonald IA, Greenhaff PL (2011). Chronic oral ingestion of L-carnitine and carbohydrate increases muscle carnitine content and alters muscle fuel metabolism during exercise in humans. J Physiol.

[6] Stephens FB, Wall BT, Marimuthu K, Shannon CE, Constantin-Teodosiu D, Macdonald IA, Greenhaff PL (2013). Skeletal muscle carnitine loading increases energy expenditure, modulates fuel metabolism gene networks and prevents body fat accumulation in humans. J Physiol.

[7] Shannon CE, Ghasemi R, Greenhaff PL, Stephens FB (2018). Increasing skeletal muscle carnitine availability does not alter the adaptations to high-intensity interval training. Scand J Med Sci Sports.

[8] Koeth RA, Wang Z, Levison BS, Buffa JA, Org E, Sheehy BT, Britt EB, Fu X, Wu Y, Li L (2013). Intestinal microbiota metabolism of L-carnitine, a nutrient in red meat, promotes atherosclerosis. Nat Med.

[9] Baltazar-Martins G, Brito de Souza D, Aguilar-Navarro M, Munoz-Guerra J, MDM P, Del Coso J (2019). Prevalence and patterns of dietary supplement use in elite Spanish athletes. J Int Soc Sports Nutr.

[10] Wardenaar FC, Ceelen IJ, Van Dijk JW, Hangelbroek RW, Van Roy L, Van der Pouw B, De Vries JH, Mensink M, Witkamp RF (2017). Nutritional supplement use by Dutch elite and sub-elite athletes: does receiving dietary counseling make a difference?. Int J Sport Nutr Exerc Metab.

[11] Wachter S, Vogt M, Kreis R, Boesch C, Bigler P, Hoppeler H, Krahenbuhl S (2002). Long-term administration of L-carnitine to humans: effect on skeletal muscle carnitine content and physical performance. Clin Chim Acta.

[12] Novakova K, Kummer O, Bouitbir J, Stoffel SD, Hoerler-Koerner U, Bodmer M, Roberts P, Urwyler A, Ehrsam R, Krahenbuhl S (2016). Effect of L-carnitine supplementation on the body carnitine pool, skeletal muscle energy metabolism and physical performance in male vegetarians. Eur J Nutr.

[13] Lohninger A, Sendic A, Litzlbauer E, Hofbauer R, Staniek H, Blesky D, Schwieglhofer C, Eder M, Bergmuller H, Mascher D (2005). Endurance exercise training and L-carnitine supplementation stimulates gene expression in the blood and muscle cells in young athletes and middle aged subjects. Monatshefte Fur Chemie.

[14] Malaguarnera M, Cammalleri L, Gargante MP, Vacante M, Colonna V, Motta M (2007). L-Carnitine treatment reduces severity of physical and mental fatigue and increases cognitive functions in centenarians: a randomized and controlled clinical trial. Am J Clin Nutr.

[15] Sawicka AK, Hartmane D, Lipinska P, Wojtowicz E, Lysiak-Szydlowska W, Olek RA. l-Carnitine Supplementation in Older Women. A Pilot Study on Aging Skeletal Muscle Mass and Function. Nutrients. 2018;10(2). 10.3390/nu10020255. 10.3390/nu10020255 PMC5852831 29473908

[16] Samulak JJ, Sawicka AK, Hartmane D, Grinberga S, Pugovics O, Lysiak-Szydlowska W, Olek RA (2019). L-Carnitine supplementation increases Trimethylamine-N-oxide but not markers of atherosclerosis in healthy aged women. Ann Nutr Metab.

[17] Olek RA, Samulak JJ, Sawicka AK, Hartmane D, Grinberga S, Pugovics O, Lysiak-Szydlowska W (2019). Increased Trimethylamine N-oxide is not associated with oxidative stress markers in healthy aged women. Oxidative Med Cell Longev.

[18] Bordoni L, Sawicka AK, Szarmach A, Winklewski PJ, Olek RA, Gabbianelli R (2020). A pilot study on the effects of l-Carnitine and Trimethylamine-N-oxide on platelet mitochondrial DNA methylation and CVD biomarkers in aged women. Int J Mol Sci.

[19] Grunewald KK, Bailey RS (1993). Commercially marketed supplements for bodybuilding athletes. Sports Med.

[20] Hawley JA, Brouns F, Jeukendrup A (1998). Strategies to enhance fat utilisation during exercise. Sports Med.

[21] Barnett C, Costill DL, Vukovich MD, Cole KJ, Goodpaster BH, Trappe SW, Fink WJ (1994). Effect of L-carnitine supplementation on muscle and blood carnitine content and lactate accumulation during high-intensity sprint cycling. Int J Sport Nutr.

[22] Vukovich MD, Costill DL, Fink WJ (1994). Carnitine supplementation: effect on muscle carnitine and glycogen content during exercise. Med Sci Sports Exerc.

[23] Rebouche CJ (1977). Carnitine movement across muscle cell membranes. Studies in isolated rat muscle. Biochim Biophys Acta.

[24] Stephens FB, Constantin-Teodosiu D, Laithwaite D, Simpson EJ, Greenhaff PL (2006). Insulin stimulates L-carnitine accumulation in human skeletal muscle. FASEB J.

[25] Stephens FB, Constantin-Teodosiu D, Laithwaite D, Simpson EJ, Greenhaff PL (2006). An acute increase in skeletal muscle carnitine content alters fuel metabolism in resting human skeletal muscle. J Clin Endocrinol Metab.

[26] Stephens FB, Evans CE, Constantin-Teodosiu D, Greenhaff PL (2007). Carbohydrate ingestion augments L-carnitine retention in humans. J Appl Physiol (1985).

[27] Attaix D, Ventadour S, Codran A, Bechet D, Taillandier D, Combaret L (2005). The ubiquitin-proteasome system and skeletal muscle wasting. Essays Biochem.

[28] Schiaffino S, Dyar KA, Ciciliot S, Blaauw B, Sandri M (2013). Mechanisms regulating skeletal muscle growth and atrophy. FEBS J.

[29] Sanchez AM, Candau RB, Bernardi H (2014). FoxO transcription factors: their roles in the maintenance of skeletal muscle homeostasis. Cell Mol Life Sci.

[30] Keller J, Ringseis R, Priebe S, Guthke R, Kluge H, Eder K (2011). Dietary L-carnitine alters gene expression in skeletal muscle of piglets. Mol Nutr Food Res.

[31] Keller J, Ringseis R, Koc A, Lukas I, Kluge H, Eder K (2012). Supplementation with l-carnitine downregulates genes of the ubiquitin proteasome system in the skeletal muscle and liver of piglets. Animal.

[32] Busquets S, Serpe R, Toledo M, Betancourt A, Marmonti E, Orpi M, Pin F, Capdevila E, Madeddu C, Lopez-Soriano FJ (2012). L-Carnitine: an adequate supplement for a multi-targeted anti-wasting therapy in cancer. Clin Nutr.

[33] Keller J, Couturier A, Haferkamp M, Most E, Eder K (2013). Supplementation of carnitine leads to an activation of the IGF-1/PI3K/Akt signalling pathway and down regulates the E3 ligase MuRF1 in skeletal muscle of rats. Nutr Metab (Lond).

[34] Keller J, Ringseis R, Eder K (2014). Supplemental carnitine affects the microRNA expression profile in skeletal muscle of obese Zucker rats. BMC Genomics.

[35] Jang J, Park J, Chang H, Lim K (2016). L-Carnitine supplement reduces skeletal muscle atrophy induced by prolonged hindlimb suspension in rats. Appl Physiol Nutr Metab.

[36] Di Marzio L, Moretti S, D'Alo S, Zazzeroni F, Marcellini S, Smacchia C, Alesse E, Cifone MG, De Simone C (1999). Acetyl-L-carnitine administration increases insulin-like growth factor 1 levels in asymptomatic HIV-1-infected subjects: correlation with its suppressive effect on lymphocyte apoptosis and ceramide generation. Clin Immunol.

[37] Kraemer WJ, Volek JS, French DN, Rubin MR, Sharman MJ, Gomez AL, Ratamess NA, Newton RU, Jemiolo B, Craig BW (2003). The effects of L-carnitine L-tartrate supplementation on hormonal responses to resistance exercise and recovery. J Strength Cond Res.

[38] Rondanelli M, Solerte SB, Fioravanti M, Scevola D, Locatelli M, Minoli L, Ferrari E (1997). Circadian secretory pattern of growth hormone, insulin-like growth factor type I, cortisol, adrenocorticotropic hormone, thyroid-stimulating hormone, and prolactin during HIV infection. AIDS Res Hum Retrovir.

[39] Evans M, Guthrie N, Pezzullo J, Sanli T, Fielding RA, Bellamine A (2017). Efficacy of a novel formulation of L-Carnitine, creatine, and leucine on lean body mass and functional muscle strength in healthy older adults: a randomized, double-blind placebo-controlled study. Nutr Metab (Lond).

[40] Askarpour M, Hadi A, Miraghajani M, Symonds ME, Sheikhi A, Ghaedi E (2020). Beneficial effects of l-carnitine supplementation for weight management in overweight and obese adults: an updated systematic review and dose-response meta-analysis of randomized controlled trials. Pharmacol Res.

[41] Lee JK, Lee JS, Park H, Cha YS, Yoon CS, Kim CK (2007). Effect of L-carnitine supplementation and aerobic training on FABPc content and beta-HAD activity in human skeletal muscle. Eur J Appl Physiol.

[42] Rafraf M, Karimi M, Jafari A (2015). Effect of L-carnitine supplementation in comparison with moderate aerobic training on serum inflammatory parameters in healthy obese women. J Sports Med Phys Fitness.

[43] Koozehchian MS, Daneshfar A, Fallah E, Agha-Alinejad H, Samadi M, Kaviani M, Kaveh BM, Jung YP, Sablouei MH, Moradi N (2018). Effects of nine weeks L-Carnitine supplementation on exercise performance, anaerobic power, and exercise-induced oxidative stress in resistance-trained males. J Exerc Nutrition Biochem.

[44] Ahlborg G, Jensen-Urstad M (1991). Metabolism in exercising arm vs. leg muscle. Clin Physiol.

[45] Doherty TJ (2003). Invited review: Aging and sarcopenia. J Appl Physiol (1985).

[46] Volpato S, Bianchi L, Cherubini A, Landi F, Maggio M, Savino E, Bandinelli S, Ceda GP, Guralnik JM, Zuliani G (2014). Prevalence and clinical correlates of sarcopenia in community-dwelling older people: application of the EWGSOP definition and diagnostic algorithm. J Gerontol A Biol Sci Med Sci.

[47] Peake J, Suzuki K (2004). Neutrophil activation, antioxidant supplements and exercise-induced oxidative stress. Exerc Immunol Rev.

[48] Peake J, Nosaka K, Suzuki K (2005). Characterization of inflammatory responses to eccentric exercise in humans. Exerc Immunol Rev.

[49] Fritz IB, Arrigoni-Martelli E (1993). Sites of action of carnitine and its derivatives on the cardiovascular system: interactions with membranes. Trends Pharmacol Sci.

[50] Giamberardino MA, Dragani L, Valente R, Di Lisa F, Saggini R, Vecchiet L (1996). Effects of prolonged L-carnitine administration on delayed muscle pain and CK release after eccentric effort. Int J Sports Med.

[51] Volek JS, Kraemer WJ, Rubin MR, Gomez AL, Ratamess NA, Gaynor P (2002). L-Carnitine L-tartrate supplementation favorably affects markers of recovery from exercise stress. Am J Physiol Endocrinol Metab.

[52] Spiering BA, Kraemer WJ, Vingren JL, Hatfield DL, Fragala MS, Ho JY, Maresh CM, Anderson JM, Volek JS (2007). Responses of criterion variables to different supplemental doses of L-carnitine L-tartrate. J Strength Cond Res.

[53] Ho JY, Kraemer WJ, Volek JS, Fragala MS, Thomas GA, Dunn-Lewis C, Coday M, Hakkinen K, Maresh CM (2010). L-Carnitine l-tartrate supplementation favorably affects biochemical markers of recovery from physical exertion in middle-aged men and women. Metabolism.

[54] Spiering BA, Kraemer WJ, Hatfield DL, Vingren JL, Fragala MS, Ho JY, Thomas GA, Hakkinen K, Volek JS (2008). Effects of L-carnitine L-tartrate supplementation on muscle oxygenation responses to resistance exercise. J Strength Cond Res.

[55] Rebouche CJ, Mack DL, Edmonson PF (1984). L-Carnitine dissimilation in the gastrointestinal tract of the rat. Biochemistry.

[56] Rebouche CJ (1991). Quantitative estimation of absorption and degradation of a carnitine supplement by human adults. Metabolism.

[57] Rebouche CJ, Chenard CA (1991). Metabolic fate of dietary carnitine in human adults: identification and quantification of urinary and fecal metabolites. J Nutr.

[58] Fukami K, Yamagishi S, Sakai K, Kaida Y, Yokoro M, Ueda S, Wada Y, Takeuchi M, Shimizu M, Yamazaki H (2015). Oral L-carnitine supplementation increases trimethylamine-N-oxide but reduces markers of vascular injury in hemodialysis patients. J Cardiovasc Pharmacol.

[59] Vallance HD, Koochin A, Branov J, Rosen-Heath A, Bosdet T, Wang Z, Hazen SL, Horvath G (2018). Marked elevation in plasma trimethylamine-N-oxide (TMAO) in patients with mitochondrial disorders treated with oral l-carnitine. Mol Genet Metab Rep.

[60] Samulak JJ, Sawicka AK, Samborowska E, Olek RA. Plasma Trimethylamine-N-oxide following Cessation of L-carnitine Supplementation in Healthy Aged Women. Nutrients. 2019;11(6). 10.3390/nu11061322. 10.3390/nu11061322 PMC6627560 31200429

[61] Wang Z, Klipfell E, Bennett BJ, Koeth R, Levison BS, Dugar B, Feldstein AE, Britt EB, Fu X, Chung YM (2011). Gut flora metabolism of phosphatidylcholine promotes cardiovascular disease. Nature.

[62] Pan A, Sun Q, Bernstein AM, Schulze MB, Manson JE, Stampfer MJ, Willett WC, Hu FB (2012). Red meat consumption and mortality: results from 2 prospective cohort studies. Arch Intern Med.

[63] Tang WH, Wang Z, Levison BS, Koeth RA, Britt EB, Fu X, Wu Y, Hazen SL (2013). Intestinal microbial metabolism of phosphatidylcholine and cardiovascular risk. N Engl J Med.

[64] Tang WH, Wang Z, Kennedy DJ, Wu Y, Buffa JA, Agatisa-Boyle B, Li XS, Levison BS, Hazen SL (2015). Gut microbiota-dependent trimethylamine N-oxide (TMAO) pathway contributes to both development of renal insufficiency and mortality risk in chronic kidney disease. Circ Res.

[65] Suzuki T, Heaney LM, Bhandari SS, Jones DJ, Ng LL (2016). Trimethylamine N-oxide and prognosis in acute heart failure. Heart.

[66] Gruppen EG, Garcia E, Connelly MA, Jeyarajah EJ, Otvos JD, Bakker SJL, Dullaart RPF (2017). TMAO is associated with mortality: impact of modestly impaired renal function. Sci Rep.

[67] Heianza Y, Ma W, Manson JE, Rexrode KM, Qi L. Gut Microbiota Metabolites and Risk of Major Adverse Cardiovascular Disease Events and Death: A Systematic Review and Meta-Analysis of Prospective Studies. J Am Heart Assoc. 2017;6(7). 10.1161/JAHA.116.004947. 10.1161/JAHA.116.004947 PMC5586261 28663251

[68] Schiattarella GG, Sannino A, Toscano E, Giugliano G, Gargiulo G, Franzone A, Trimarco B, Esposito G, Perrino C (2017). Gut microbe-generated metabolite trimethylamine-N-oxide as cardiovascular risk biomarker: a systematic review and dose-response meta-analysis. Eur Heart J.

[69] Rebouche CJ, Engel AG (1984). Kinetic compartmental analysis of carnitine metabolism in the human carnitine deficiency syndromes. Evidence for alterations in tissue carnitine transport. J Clin Invest.

[70] Wang Z, Bergeron N, Levison BS, Li XS, Chiu S, Jia X, Koeth RA, Li L, Wu Y, Tang WHW (2019). Impact of chronic dietary red meat, white meat, or non-meat protein on trimethylamine N-oxide metabolism and renal excretion in healthy men and women. Eur Heart J.

[71] Rohrmann S, Linseisen J, Allenspach M, von Eckardstein A, Muller D (2016). Plasma concentrations of Trimethylamine-N-oxide are directly associated with dairy food consumption and low-grade inflammation in a German adult population. J Nutr.

[72] Cheung W, Keski-Rahkonen P, Assi N, Ferrari P, Freisling H, Rinaldi S, Slimani N, Zamora-Ros R, Rundle M, Frost G (2017). A metabolomic study of biomarkers of meat and fish intake. Am J Clin Nutr.

[73] Yancey PH, Clark ME, Hand SC, Bowlus RD, Somero GN (1982). Living with water stress: evolution of osmolyte systems. Science.

[74] Gillett MB, Suko JR, Santoso FO, Yancey PH (1997). Elevated levels of trimethylamine oxide in muscles of deep-sea gadiform teleosts: a high-pressure adaptation?. J Exp Zool.

[75] Yancey PH, Gerringer ME, Drazen JC, Rowden AA, Jamieson A (2014). Marine fish may be biochemically constrained from inhabiting the deepest ocean depths. Proc Natl Acad Sci U S A.

[76] Zhang AQ, Mitchell SC, Smith RL (1999). Dietary precursors of trimethylamine in man: a pilot study. Food Chem Toxicol.

[77] Tong TYN, Appleby PN, Bradbury KE, Perez-Cornago A, Travis RC, Clarke R, Key TJ (2019). Risks of ischaemic heart disease and stroke in meat eaters, fish eaters, and vegetarians over 18 years of follow-up: results from the prospective EPIC-Oxford study. BMJ.

[78] Bain MA, Faull R, Fornasini G, Milne RW, Evans AM (2006). Accumulation of trimethylamine and trimethylamine-N-oxide in end-stage renal disease patients undergoing haemodialysis. Nephrol Dial Transplant.

[79] Hauet T, Baumert H, Gibelin H, Godart C, Carretier M, Eugene M (2000). Citrate, acetate and renal medullary osmolyte excretion in urine as predictor of renal changes after cold ischaemia and transplantation. Clin Chem Lab Med.

[80] Gansevoort RT, Correa-Rotter R, Hemmelgarn BR, Jafar TH, Heerspink HJ, Mann JF, Matsushita K, Wen CP (2013). Chronic kidney disease and cardiovascular risk: epidemiology, mechanisms, and prevention. Lancet.

[81] Damman K, Valente MA, Voors AA, O'Connor CM, van Veldhuisen DJ, Hillege HL (2014). Renal impairment, worsening renal function, and outcome in patients with heart failure: an updated meta-analysis. Eur Heart J.

[82] Bielinska K, Radkowski M, Grochowska M, Perlejewski K, Huc T, Jaworska K, Motooka D, Nakamura S, Ufnal M (2018). High salt intake increases plasma trimethylamine N-oxide (TMAO) concentration and produces gut dysbiosis in rats. Nutrition.

[83] Jaworska K, Huc T, Samborowska E, Dobrowolski L, Bielinska K, Gawlak M, Ufnal M (2017). Hypertension in rats is associated with an increased permeability of the colon to TMA, a gut bacteria metabolite. PLoS One.

[84] Xu M, Bhatt DK, Yeung CK, Claw KG, Chaudhry AS, Gaedigk A, Pearce RE, Broeckel U, Gaedigk R, Nickerson DA (2017). Genetic and nongenetic factors associated with protein abundance of Flavin-containing Monooxygenase 3 in human liver. J Pharmacol Exp Ther.

[85] Ufnal M, Pham K (2017). The gut-blood barrier permeability - a new marker in cardiovascular and metabolic diseases?. Med Hypotheses.

[86] Lango R, Smolenski RT, Narkiewicz M, Suchorzewska J, Lysiak-Szydlowska W (2001). Influence of L-carnitine and its derivatives on myocardial metabolism and function in ischemic heart disease and during cardiopulmonary bypass. Cardiovasc Res.

[87] Iliceto S, Scrutinio D, Bruzzi P, D'Ambrosio G, Boni L, Di Biase M, Biasco G, Hugenholtz PG, Rizzon P (1995). Effects of L-carnitine administration on left ventricular remodeling after acute anterior myocardial infarction: the L-Carnitine Ecocardiografia Digitalizzata Infarto Miocardico (CEDIM) trial. J Am Coll Cardiol.

[88] Hiramatsu A, Aikata H, Uchikawa S, Ohya K, Kodama K, Nishida Y, Daijo K, Osawa M, Teraoka Y, Honda F (2019). Levocarnitine use is associated with improvement in sarcopenia in patients with liver cirrhosis. Hepatol Commun.

[89] Hathcock JN, Shao A (2006). Risk assessment for carnitine. Regul Toxicol Pharmacol.

[90] Shang R, Sun Z, Li H (2014). Effective dosing of L-carnitine in the secondary prevention of cardiovascular disease: a systematic review and meta-analysis. BMC Cardiovasc Disord.

[91] Bakalov D, Sabit Z, Tafradjiiska-Hadjiolova R (2020). Re: effect of l-carnitine supplementation on muscle cramps induced by stroke: a case report. Nutrition.

